# Psychosocial Predictors of Maternal–Fetal Attachment in Anxious Pregnancies: A Study Among Women With Prior Miscarriages

**DOI:** 10.1002/hsr2.72470

**Published:** 2026-04-29

**Authors:** Iqra Javaid, Muhammad Ameeq, Muhammad Muneeb Hassan, Alpha Kargbo, Muhammad Daud Butt, Saadia Zia

**Affiliations:** ^1^ Punjab Thalassaemia & Other Genetic Disorders Prevention and Research Institute Bahawalpur Victoria Hospital Bahawalpur Pakistan; ^2^ Department of Statistics TheIslamia University Bahawalpur Pakistan; ^3^ Multimedia University, Jalan Ayer Keroh Lama Melaka Malaysia; ^4^ Department of Physical & Natural Science University of the Gambia Serekunda Gambia; ^5^ School of Pharmaceutical Sciences Universiti Sains Malaysia Penang USM Malaysia; ^6^ Department of Psychology University of Southern Punjab Multan Pakistan

**Keywords:** anxiety, mental health, miscarriages, parental depression, pregnant women

## Abstract

**Background:**

Maternal mental health influences the key psychological process of maternal–fetal attachment (MFA) during pregnancy. Women with a history of miscarriage are at increased risk of prenatal anxiety and depression, which may affect their emotional bond with the fetus. Evidence on how anxiety and depression influence MFA in high‐risk pregnancies is inconsistent, particularly in low‐income settings such as Pakistan, where prenatal mental health research is limited.

**Aims:**

This study aimed to examine the association between prenatal anxiety and prenatal depression with maternal–fetal attachment and its dimensions among pregnant women with a history of miscarriage and to explore differences based on selected demographic and psychosocial factors.

**Methods:**

A cross‐sectional study was conducted at Bahawalpur Victoria Hospital, Pakistan, from October 2024 to December 2024. A total of 523 third‐trimester pregnant women with prior miscarriages were recruited using purposive sampling. Prenatal depression, anxiety, and maternal–fetal attachment were assessed using the EPDS, SAS, and MAAS, respectively. Independent sample *t*‐tests, Pearson's correlations, and multiple linear regression analyses were performed using SPSS 27.0.

**Results:**

Prenatal anxiety was significantly and positively associated with maternal–fetal attachment and its subdimensions, whereas prenatal depression showed no such association. Regression analysis confirmed anxiety as a significant predictor of attachment quality and total MFA scores, whereas depression was non‐significant. Planned pregnancy, marital satisfaction, employment, sleep disturbances, and younger maternal age were associated with stronger maternal–fetal attachment and higher anxiety.

**Conclusion:**

Prenatal anxiety, but not depression, is positively associated with maternal–fetal attachment among women with a history of miscarriage. These findings emphasize the importance of the early identification and management of prenatal anxiety and highlight the need for culturally appropriate mental health interventions to support maternal well‐being and mother–fetus bonding in high‐risk pregnancies.

## Summary

This study suggests that anxiety, but not depression, affects the mother–fetus bond in women with high‐risk pregnancies, highlighting the significance of assessing prenatal anxiety during pregnancy to develop a strong maternal–fetal bond.

**Abbreviations**
BVH: Bahawalpur Victoria Hospital
CI: confidence interval
EPDS: Edinburgh postnatal depression scale
GLM: generalized linear model
IQR: interquartile range
IRB: institutional review board
MAAS: maternal antenatal attachment scale
MFA: maternal–fetal attachment
PRA: pregnancy related anxiety
SAS: Zung self‐rating anxiety scale
SD: standard deviation
SPSS: statistical package for the social science

## 1 Introduction

The transition to motherhood, characterized by profound psychological and physiological changes [1], presents considerable challenges for many women, who are particularly vulnerable to experiencing prenatal depression and anxiety [2, 3]. This vulnerability is often intensified in women with a history of miscarriage [4], who may experience significant emotional distress [2], potentially compromising maternal–fetal attachment (MFA) [3]. MFA refers to the emotional bond [5] that develops between a pregnant woman and her unborn child during gestation [6] and is widely acknowledged as a fundamental precursor to postpartum bonding [7], maternal physical attachment [8], and early caregiving behaviors [9].

Recurrent pregnancy loss is consistently associated with increased depression, chronic stress [2], low self‐esteem, hopelessness, confusion, and frustration [10]. Persistent prenatal depression and anxiety can result in prolonged psychological distress and impaired emotional functioning [11]. Therefore, a comprehensive understanding of these psychological dimensions is crucial for optimizing maternal mental health [12], particularly among women who conceive following pregnancy loss [13].

Experiencing multiple miscarriages is widely acknowledged as a profoundly stressful life event, often characterized by emotional fragility, reduced self‐worth, and an ongoing fear of fetal loss, which may disrupt early prenatal bonding [14, 15]. These emotional challenges may be further exacerbated by low socioeconomic status or inadequate spousal support [16, 17]. Therefore, early prenatal mental health interventions have the potential to improve maternal psychological well‐being [18] and reduce depressive symptoms among vulnerable groups of women [19].

The existing literature presents mixed findings regarding the association between maternal psychological well‐being and MFA. Studies have reported that women experiencing prenatal depression demonstrate weaker maternal–fetal attachment [20] and reduced emotional attachment to their unborn child [21, 22]. A systematic review by Rollè et al. found a negative relationship between prenatal depression and MFA, particularly in high‐risk pregnancies and culturally diverse populations [22]. While a few past studies [23, 24, 25] did not show an association between prenatal depression and some dimensions of antenatal attachment. These inconsistencies suggest that although depression is prevalent during pregnancy [1, 2, 3, 15], it does not uniformly impair maternal–fetal bonding [22], especially in contexts shaped by heightened risk or cultural variation.

Prenatal anxiety also appears to have a nuanced and multifaceted relationship with MFA. Research indicates that this association varies according to the type of anxiety experienced [26] and the specific dimensions of attachment, such as quality or intensity, as measured by the Maternal Antenatal Attachment Scale (MAAS) [27]. Women with a history of prenatal loss often report significantly higher anxiety levels [28], which have been associated with reduced perceived emotional closeness during subsequent pregnancies [29]. This type of psychological distress can persist for several months during pregnancy, increasing fear and apprehension as the pregnancy progresses [29]. On the other hand, anxiety has been associated with heightened vigilance, protective behaviors, and greater cognitive and emotional involvement with the fetus [30]. Although anxiety is more common in early pregnancy, these adaptive responses may enhance certain aspects of maternal–fetal attachment over time [31]. This apparent paradox indicates that anxiety can differentially influence maternal–fetal attachment by reducing emotional quality while simultaneously increasing maternal preoccupation and involvement with the unborn child [32].

Although most research on prenatal psychological well‐being has focused on high‐income countries [33], exciting evidence indicates that low‐and middle‐income countries, such as Pakistan, India, and Bangladesh, experience a substantial burden of prenatal distress, particularly depression and anxiety [34, 35]. Despite the high prevalence, there is limited empirical understanding of the psychological experiences of Pakistani women during high‐risk pregnancies, particularly after miscarriage. Addressing this gap is essential for advancing trauma‐informed care, improving prenatal mental health services, and developing culturally sensitive screening programs and targeted interventions for this vulnerable population. Accordingly, the present study aimed to examine the relationships between prenatal anxiety, prenatal depression, and maternal–fetal attachment and its dimensions, as well as to compare levels of attachment, anxiety, and depression among women with a history of miscarriage, across selected demographic and psychosocial factors.

Hypothesis

Women with higher anxiety will have stronger maternal–fetal attachment and its dimensions.

Prenatal depression will not show a significant association with maternal–fetal attachment or its dimensions.

Women with planned pregnancies will show stronger maternal–fetal attachment than those with unplanned pregnancies.

Women who are satisfied with their marriage will have stronger maternal–fetal attachment and lower depression.

Employed women will have higher anxiety and stronger maternal–fetal attachment than unemployed women.

Women with sleep problems will have higher anxiety and stronger maternal–fetal attachment.

Younger women (15–30 years) will have higher anxiety and stronger maternal–fetal attachment than older women (31–45 years).

## 2 Methodology

This study was conducted at Bahawal Victoria Hospital (BVH), a tertiary care facility located in South Punjab, Pakistan. Data were collected during the fiscal year of 2024 and the months of August–December. Eligible participants met the following inclusion criteria: third trimester of a singleton pregnancy, aged 15–45 years, and a history of miscarriage. Women experiencing their first pregnancy (primigravida) or those who were previously diagnosed with anxiety disorders were excluded from the study. Participants were selected using purposive sampling methods. A total of 523 questionnaires were collected, and incomplete responses were excluded from the final analysis.

### 2.1 Ethical Considerations

The study was conducted in accordance with the ethical standards of the Institutional Research Committee (IRC) and the 1964 Helsinki Declaration. The departmental review committee of Bahawalpur Victoria Hospital approved this study. Data were gathered from adult volunteers in the South Punjab region (Bahawalpur) and were completely voluntary. All participants provided informed consent after being fully informed of the study's purpose, and strict confidentiality was ensured.

### 2.2 Demographic and Pregnancy‐Related Characteristics

A structured, self‐designed questionnaire was used to gather demographic and pregnancy‐related information. The variables included gestational age, maternal age, education level, parity, pregnancy planning status, perception of body image changes, prenatal education participation, employment status during pregnancy, marital satisfaction, and sleep disturbances. All items except age, education, gestational age, and parity were assessed using binary (yes/no) questions. Examples include “Did you plan this pregnancy?”, “Do you accept body shape changes during pregnancy?” and “Have you attended a standardized prenatal education course?”

### 2.3 Assessment Instrument

#### 2.3.1 Prenatal Depression

The Edinburgh Postnatal Depression Scale (EPDS), a validated 10‐item self‐report tool [35], was used to assess prenatal depression symptoms in this study. Each item is scored from 0 to 3, yielding a total score ranging from 0 to 30. A score of ≥ 12 was considered indicative of significant prenatal depression, whereas a score of ≤ 12 was classified as the absence of prenatal depression. The EPDS has been widely used for decades and has demonstrated good reliability and validity.

#### 2.3.2 Maternal–Fetal Attachment (MFA)

The Maternal Antenatal Attachment Scale (MAAS), a 19‐item self‐report measure rated on a 5‐point Likert scale [36], was used to assess maternal–fetal attachment. The scale comprises two subscales: MAAS‐intensity of preoccupation, which measures the amount of effort and time a mother devotes to thinking about the unborn child, and MAAS‐quality of attachment, which reflects the mother's emotional experience and feelings toward her fetus.

#### 2.3.3 Prenatal Anxiety

The Zung Self‐Rating Anxiety Scale (SAS), a 20‐item self‐report questionnaire, was used to assess prenatal anxiety [37]. We assigned each item a score from 1 to 4, and then converted the total scores into standard scores by multiplying them by 1.25. A standard score of 50 or greater was considered a sign of strong anxiety, with higher values indicating more severe anxiety symptoms.

### 2.4 Data Analysis

In this study, the normality of the continuous variables was tested prior to analysis using the Shapiro–Wilk test. Normality testing was interpreted, and parametric test analysis was performed to avoid slight variations due to the large sample size. Collected data were analyzed using the Statistical Package for the Social Sciences (SPSS) 27.0 version, applying independent *t*‐test, Pearson's correlation, and multiple linear regression to check the hypothesis.

## 3 Results

### 3.1 Descriptive Statistics

Descriptive statistics were used to test the variability of the maternal–fetal attachment and psychological well‐being, as presented in (Table 1). The EPDS scores were (*M* = 12.66 and SD = 3.72), indicating that the majority of women experienced depression during pregnancy. Similarly, the anxiety score (SAS) was (*M* = 45.06, SD = 6.13), reflecting mild‐to‐severe anxiety levels among women. Furthermore, it was determined that the mean score of the MAAS subscale of “attachment quality” was (*M* = 36.48, SD = 6.10), and the mean score of the MAAS's subscale of the “intensity of preoccupation” was (*M* = 19.46, SD = 2.62). In addition, the total MAAS score had a mean of (*M* = 55.94, SD = 7.88), suggesting that the majority of women had strong fetal attachment despite the fear of prior miscarriage.

**Table 1:** Descriptive statistics of EPDS, SAS, MAAS quality of attachment, MAAS intensity of preoccupation, and total MAAS scores.

Variables	*N*	Minimum	Maximum	Mean	SD
EPDS score	523	4	26	12.66	3.274
SAS score	523	33	58	45.06	6.127
MAAS quality	523	23	47	36.48	6.104
MAAS intensity	523	14	25	19.46	2.615
Total MAAS	523	37	67	55.94	7.880

### 3.2 Group Differences in Depression, Anxiety, and Maternal–Fetal Attachment

Differences in maternal psychological well‐being and maternal‐fetal attachment were investigated using independent samples *t*‐tests. The comparison analyses considered various sociodemographic and psychosocial variables, including planned pregnancy, employment status, marital satisfaction, sleep disturbances, and age group.

### 3.3 Planned Pregnancy

Of the 523 women who participated, a significant difference was observed between those with planned pregnancies (*n* = 230) and those with unplanned pregnancies (*n* = 292). It was determined that women with unplanned pregnancies frequently had high levels of depressive symptoms (*M* = 13.29, SD = 2.73) compared to those with planned pregnancies (*M* = 11.87, SD = 3.71) (*t *= −5.02, *p* < 0.001, *d* = 3.20). However, women with planned pregnancies (*M* = 46.63, SD = 5.73) had higher levels of anxiety than those with unplanned pregnancies (*M* = 43.79, SD = 6.13) (*t *= −5.41, *p* < 0.015, *d* = 5.96). Furthermore, significant differences were observed in the maternal–fetal attachment. Women with planned pregnancies had higher scores on the MAAS Quality of Attachment, MAAS intensity of preoccupation, and total attachment compared to those with unplanned pregnancies, with large effect sizes (Cohen's *d* > 2), as shown in Table 2.

**Table 2:** Plan pregnancy comparison through an independent sample *t*‐test among EPDS score, SAS score, MAAS quality, MAAS intensity, and total MAAS.

Plan pregnancy Yes *n* = 230 No *n* = 292									
	*M*	SD	*M*	SD	*T*	*p*	95% CI		Cohen's *d*
							LL	UL	
EPDS score	11.87	3.71	13.29	2.73	−5.024	0.000	−1.97	−0.86	3.20
SAS score	46.63	5.73	43.79	6.13	5.41	0.015	1.81	3.88	5.6
MAAS quality	40.80	3.53	33.06	5.51	18.48	0.000	6.91	8.56	4.75
MAAS intensity	20.79	2.40	18.40	2.27	11.60	0.025	1.98	2.79	2.33
Total MAAS	61.58	4.28	51.46	7.17	18.91	0.000	9.07	11.17	6.07

Abbreviations: *M *= mean, *N* = 523, SD = standard deviation.

### 3.4 Marital Satisfaction

Women with satisfied marriages showed significantly better maternal–fetal attachment or emotional bonding compared to unsatisfied marriages (Table 3). Specifically, women satisfied with their relationship had lower EPDS scores than dissatisfied women (*t *= −7.06, *p *< 0.001). Although we found that anxiety (SAS) scores were higher among women having marital satisfaction, this difference did not reach statistical significance (*t* = 7.65, *p *= 0.077). In addition, the satisfied women reported higher MAAS Quality (*M* = 41.10 vs. 32.02), MAAS Intensity (*M* = 21.26 vs. 17.72), and total MAAS scores (*M* = 62.36 vs. 49.73), all with large effect sizes (*d* > 4), and all with statistically significant differences (*p*< 0.001).

**Table 3:** Marriage satisfaction comparison through an independent sample *t*‐test among EPDS score, SAS score, MAAS quality, MAAS intensity, and total MAAS.

Variables	Marital satisfaction (yes) *n* = 257		Marital satisfaction (no) *n* = 266		*t*‐value	*p* value	95% CI LL	95% CI UL	Cohen's *d*
	*M* (mean)	SD	*M* (mean)	SD					
EPDS score	11.68	3.480	13.61	2.752	−7.055	0.000	−2.470	1.394	3.131
SAS score	47.04	5.760	43.15	5.868	7.645	0.077	2.889	4.888	5.815
MAAS quality	41.10	3.009	32.02	4.890	25.487	0.000	8.386	9.781	4.076
MAAS intensity	21.26	2.376	17.72	1.357	21.005	0.025	3.208	3.870	1.926
Total MAAS	62.36	3.518	49.73	5.633	30.620	0.000	11.815	13.435	4.721

Abbreviations: *M *= mean, *N* = 523, SD = standard deviation.

### 3.5 Employment Status

The comparison based on employment, as presented in Table 4, showed that employed women experienced more depressive symptoms (*M* = 13.91, SD = 2.64) with a history of miscarriages than unemployed women, but the differences between the groups were not statistically significant (*p* > 0.05).

**Table 4:** Employed women comparison through an independent sample *t*‐test among EPDS score, SAS score, MAAS quality, MAAS intensity, and total MAAS.

Variables	Employed (yes) *n* = 215		Employed (no) *n* = 308		*t*‐value	*p* value	95% CI LL	95% CI UL	Cohen's *d*
	*M* (mean)	SD	*M* (mean)	SD					
EPDS score	13.91	2.64	11.79	3.39	6.672	0.399	1.576	2.660	3.106
SAS score	46.65	5.57	43.94	6.25	5.088	0.001	1.661	3.751	5.986
MAAS quality	40.49	4.15	33.68	5.67	15.001	0.000	5.915	7.698	5.106
MAAS intensity	21.14	1.79	18.28	2.45	15.571	0.027	2.472	3.242	2.206
Total MAAS	61.63	4.31	51.96	7.35	17.298	0.000	8.566	10.761	6.286

Abbreviations: *M* = mean, *N* = 523, SD = standard deviation.

Similarly, employed women experienced higher levels of anxiety (*M* = 46.65, SD = 5.57), stronger emotional attachment (*M* = 40.49, SD = 4.15), greater preoccupation with the fetus (*M* = 21.14, SD = 1.79), and higher total attachment scores (*M* = 61.63, SD = 4.31), all with (*p* < 0.05).

### 3.6 Sleep Disturbance

Women with sleep disturbances reported more prenatal anxiety (*M *= 45.78, SD = 5.08), (*t *= 1.98, *p *= 0.000) and statistically significant (Table 5), whereas differences in depression levels (*M* = 13.51, SD = 3.15). Scores indicate that women with a sleeping problem during pregnancy showed significantly stronger maternal–fetal attachment and all subscales (all *p* < 0.01), indicating high emotional bonding and cognitive involvement with the fetus.

**Table 5:** Sleep disorder comparison through an independent sample *t*‐test among EPDS score, SAS score, MAAS quality, MAAS intensity, and total MAAS.

Variables	Sleep disorder (yes) *n* = 182		Sleep disorder (no) *n* = 341		*t*‐value	*p* value	95% CI LL	95% CI UL	Cohen's *d*
	*M* (mean)	SD	*M* (mean)	SD					
EPDS score	13.51	3.15	12.21	3.25	4.391	0.413	0.717	1878	3.218
SAS score	45.78	5.08	44.67	6.59	1.977	0.000	0.007	2.211	6.110
MAAS quality	38.24	3.62	35.54	6.91	4.913	0.000	1.617	3.771	5.973
MAAS intensity	20.65	2.68	18.82	2.35	8.065	0.002	1.382	2.272	2.468
Total MAAS	58.88	5.12	54.36	8.62	6.492	0.000	3.153	5.889	7.586

Abbreviations: *M *= mean, *N* = 523, SD = standard deviation.

### 3.7 Age Group

There is no significant difference in EPDS scores between the two age groups, 15–30 years (*n* = 241) and 31–45 years (*n *= 282), as shown in Table 6. However, younger women reported significantly higher anxiety (*M* = 47.15, SD = 5.421), greater MAAS attachment of quality (*M* = 37.20, SD = 4.1), and higher MAAS intensity of preoccupation (*M* = 20.21, SD = 18.82), all *p* < 0.001, suggesting that younger women may experience stronger emotional attachment and fetal bonding but also have more anxiety during pregnancy.

**Table 6:** Age comparison through an independent sample *t*‐test among EPDS score, SAS score, MAAS quality, MAAS intensity, and total MAAS.

Variables	Age (15–30) years *n* = 241		Age (31–45) years *n* = 282		*t*‐value	*p* value	95% CI LL	95% CI UL	Cohen's *d*
	*M* (mean)	SD	*M* (mean)	SD					
EPDS score	12.69	3.960	12.63	2.553	0.215	0.000	−0.503	0.626	3.277
SAS score	47.15	5.421	43.27	6.138	7.601	0.000	2.877	4.883	5.818
MAAS quality	37.20	4.120	35.87	7.341	2.489	0.000	0.280	2.373	6.073
MAAS intensity	20.21	2.669	18.82	2.392	6.287	0.000	0.957	1.827	2.524
Total MAAS	57.40	5.787	54.68	9.128	3.988	0.000	1.379	4.057	7.770

Abbreviations: *M* = mean, *N* = 523, SD = standard deviation.

### 3.8 Correlation Among the Anxiety, Depression and MFA Dimension Variables

**Figure 1:**
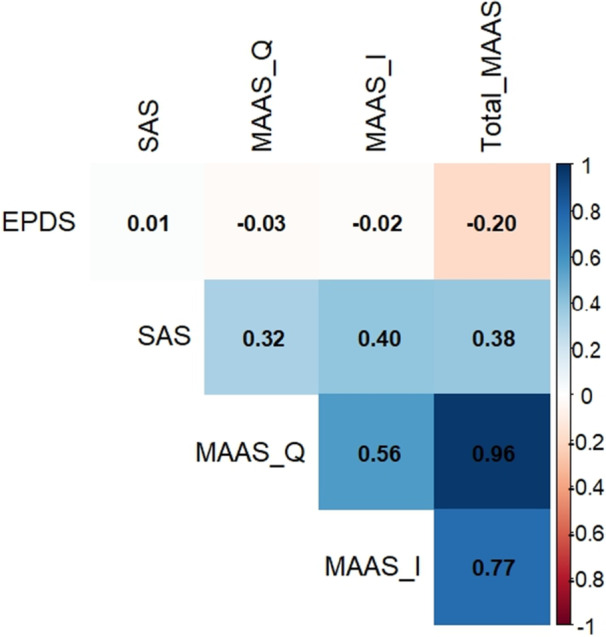
Correlation heatmap of psychosocial predictors.

Pearson's correlation analysis was conducted to explore the correlations shown in Table 7 and graphically presented in Figure 1. The analysis determined that the EPDS scores were not significantly correlated with any aspect of prenatal attachment. In contrast, anxiety was positively correlated with all three aspects of prenatal attachment, including MAAS quality of attachment (*r* = 0.321, *p* < 0.01), MAAS intensity of preoccupation (*r* = 0.397, *p* < 0.01), and total MAAS scores (*r* = 0.381, *p* < 0.01). It was also determined that there were strong intercorrelations among the MAAS subscales, particularly between the intensity of preoccupation(*r* = 0.564, *p* < 0.01) and total MAAS (*r* = 0.962, *p* < 0.01).

**Table 7:** Correlation among the EPDS, SAS, MAAS quality of attachment, MAAS intensity of preoccupation, and total MAAS Scores.

Variables	1	2	3	4	5
EPDS score	1	0.010	−0.025	−0.020	−0.26
SAS score			0.321*	0.397*	0.381*
MAAS quality				0.564*	0.962*
MAAS intensity					0.769*
Total MAAS					

* Correlations significant at the 0.01 level (two‐tailed).

### 3.9 Regression Analysis of Maternal Mental Health and Prenatal Attachment

Multiple linear regression analyses were conducted to predict how depressive symptoms (EPDS scores) and anxiety levels (SAS scores) influenced the MAAS quality of attachment, MAAS intensity of preoccupation with the fetus, and the total MAAS (overall maternal–fetal bonding).

Anxiety (SAS scores) significantly predicted attachment quality (*β *= 0.322, *t* = 7.75, *p* < 0.001), demonstrating that higher anxiety levels were linked to stronger attachment quality. However, depression (EPDS scores) was not a significant predictor (*β *= −0.028, *p *= 0.498), as shown in Table 8.

**Table 8:** Regression model of EPDS, SAS score, and quality of attachment.

	**Unstandardized coefficients**		**Standardized coefficients**		
**Model**	***B***	**Std. error**	**Beta**	***t***	**Sig**
(Constant)	22.701	2.112	—	10.749	0.000
EPDS score	−0.052	0.077	−0.028	−0.678	0.498
SAS score	0.321	0.041	0.322	7.752	0.000

*Note:* Dependent variable: quality of attachment.

Notably, both maternal depression and anxiety had no significant effects on the intensity of preoccupation. As shown in Table 9, neither EPDS (*β* = −0.023, *p *= 0.561) nor SAS scores (*β* = −0.028, *p *= 0.498) were significant predictors of the intensity of preoccupation.

**Table 9:** Regression model of EPDS, SAS score, and intensity of preoccupation.

	Unstandardized coefficients		Standardized coefficients		
Model	*B*	Std. error	Beta	*t*	Sig
(Constant)	12.057	0.877	—	13.744	0.000
EPDS score	−0.019	0.032	−0.023	−0.582	0.561
SAS score	−0.052	0.077	−0.028	−0.678	0.498

*Note:* Dependent variable: intensity of preoccupation.

In Table 10, EPDS scores remained non‐significant (*β* = −0.030, *p *= 0.466), and SAS scores were again a significant positive predictor of total MAAS scores (*β* = 0.381, *t* = 9.40, *p*< 0.001). These findings suggest that anxiety, but not depression, is positively associated with overall maternal–fetal attachment.

**Table 10:** Regression model of EPDS, SAS score, and total MAAS.

	Unstandardized coefficients		Standardized coefficients		
Model	*B*	Std. error	Beta	*t*	Sig
(Constant)	34.758	2.662	—	13.056	0.000
EPDS Score	−0.071	0.098	−0.030	−0.730	0.466
SAS Score	0.490	0.052	0.381	9.401	0.000

*Note:* dependent variable: total MAAS.

## 4 Discussion

The present study aimed to evaluate the role of maternal psychological well‐being with respect to maternal–fetal attachment among women with a previous miscarriage history. The findings revealed a significant correlation between prenatal anxiety and maternal–fetal attachment or its dimensions, while no significant correlation was observed between prenatal depression and maternal–fetal attachment or its dimensions, despite the high depression score in high‐risk pregnancies. In addition, different contextual variables, including sleep disturbance, planned pregnancy, marriage satisfaction, employment status, and maternal age, were also shown to have significant associations related to maternal psychological well‐being and maternal–fetal attachment in our study population.

According to our results, a significant positive relationship was reported between prenatal anxiety and maternal–fetal attachment, particularly with quality of attachment, intensity of preoccupations, and total scores (*r *= 0.321–0.962, *p *< 0.01), indicating that prenatal anxiety was a strong predictor of maternal–fetal attachment in high‐risk pregnancies. Göbel et al. found that women with a history of miscarriage have higher levels of anxiety, which may correlate with impaired development of a healthy mother–child relationship [27]. According to Brockington et al. and O'Dea et al., fear of pregnancy loss may enhance mother‐emotional bonding by intensifying attentional focus on fetal health, thereby strengthening certain dimensions of attachment [38, 39]. Our findings are consistent with earlier evidence suggesting that anxiety in high‐risk pregnancies can coexist with heightened maternal involvement and preoccupation with the unborn child [31]. This interpretation will indicate that prenatal anxiety may differentially influence maternal–fetal attachment and its dimensions, increasing maternal vigilance and preoccupation while not always reflecting optimal emotional quality of attachment [26, 27, 40]. On the other hand, prenatal depression in this study was reported to be not significantly associated with maternal–fetal attachment or any of its dimensions. Even though symptoms of depression were prevalent in our participants, regression and correlation analyses showed that women who experience prenatal depression did not actually predict attachment quality, intensity of preoccupations, and overall maternal–fetal attachment scores. This is consistent with past literature on pregnant women with high‐risk pregnancies, where no significant association between prenatal depression and MFA [41, 42, 43, 44]. Past studies suggest that depression diminishes maternal emotional bonding and intimacy with the fetus [45, 46]; however, evidence remains mixed, particularly among women having a history of miscarriage. This pattern may reflect that various contextual or cultural factors in the study population can impact prenatal anxiety and depression to further intensify maternal vigilance and engagement with the fetus [47].

Our findings also demonstrated that various demographic and psychological factors show a relationship with maternal psychological well‐being and maternal–fetal attachment. Women with planned pregnancies and satisfied marriages, compared to those with unplanned or unsatisfied marriages, report significantly lower depressive symptoms and higher anxiety levels, alongside stronger maternal–fetal attachment. This may highlight that healthy family relationships reduce depression but may simultaneously increase anxiety among women due to heightened emotional investment or fear of losing their pregnancy [48]. Stefana et al. reported a similar result where lack of partner support and communication undermines the personal well‐being of the mother [49]. Managing strong maternal–fetal attachment in high‐risk pregnancies is challenging for many Pakistani women; therefore, family, partner, and healthcare support may help in alleviating maternal anxiety [50]. Similarly, employed women showed higher anxiety alongside greater maternal–fetal attachment; therefore, employment status is a critical determinant of psychological well‐being. A similar relationship was also reported in women with sleep disturbance, where sleep deprivation and work‐related stress exacerbate emotional stress during pregnancy [51, 52]. Couto et al. reported that women experiencing sleep insufficiency or job strain during pregnancy exhibited higher depressive symptoms and anxiety [53]. Prenatal depression and anxiety in younger mothers (15–30 years) were higher compared to older mothers (31–45 years). This suggests that psychological resilience and life experience with age can impact the mental health and well‐being of pregnant women [54].

To the best of our knowledge, the strength of the current study is that no research has evaluated the relationship between prenatal anxiety and depression with respect to maternal–fetal attachment in high‐risk pregnancies of Pakistani women. Concerning the limitations, firstly, not including longitudinal follow‐up makes the finding ungeneralizable. Second, women recruited for this cross‐sectional study from a single geographical region of Pakistan limit the reliability of the study. Therefore, we strongly recommend recruiting a large sample size for longitudinal or mixed study designs to further investigate mothers' feelings and life experiences during high‐risk pregnancies for future studies.

### 4.1 Study Implications

Our study findings underscore the complicated impact of a history of miscarriage on women's psychological well‐being and maternal‐fetal attachment during pregnancy. To address barriers related to mental health support for women with a history of miscarriage, it is important to identify psychological and demographic variables associated with maternal‐fetal attachment. Hence, our findings assist mental health support workers and policymakers in integrating culturally sensitive support interventions in low‐income countries.

## 5 Conclusion

The finding indicates that women with a history of miscarriage are more vulnerable to prenatal anxiety and depression, undermining the critical need for maternal mental health support. Key findings also show that marital satisfaction, sleep quality, employment status, planned pregnancy, and the age of the mother are significantly correlated with maternal‐fetal attachment and maternal mental health. Prenatal anxiety demonstrated a strong correlation with maternal‐fetal attachment, indicating increased emotional involvement and attachment to the fetus. Based on these findings, implementing psychological counseling programs, prenatal education with peer support groups, and enhanced workplace policies for employed women could provide substantial support for the mental health of women with a history of miscarriages. It is essential for healthcare professionals and policymakers to prioritize prenatal care strategies that foster maternal mental health and strengthen maternal–fetal bonding.

### 5.1 Data Quality and Instrument Validation

Rigorous translation in Urdu (local language) was done to ensure conceptual equivalence and the validity of the data collection and measurement scale used in our study. We used EPDS, MAAS, and SAS scales, which are internationally validated tools for assessing mental health. The demographic questionnaire was reviewed by an expert in psychology and mental health. Prior to research, a pilot test was conducted.

## Author Contributions

**Iqra Javaid:** conceptualization, data curation, and investigation. **Muhammad Ameeq:** investigation, data analysis, writing original draft, writing – review and editing. **Muhammad Muneeb Hassan:** software, data analysis, and conceptualization. **Alpha Kargbo:** software, data analysis, conceptualization, data collection, and data cleaning. **Muhammad Daud Butt:** data analysis, writing original draft, writing – review and editing. **Saadia Zia:** investigation and data curation.

## Funding

The authors have nothing to report.

## Ethics Statement

This study was conducted in accordance with the ethical standards of the Institutional Research Committee and the 1964 Helsinki Declaration and its subsequent amendments. Ethical approval was obtained from the Ethics Review Committee of Bahawalpur Victoria Hospital prior to the commencement of the study.

## Consent

All participants provided written informed consent after being fully briefed on the study's purpose, procedures, and confidentiality of their responses. Participation was entirely voluntary, and the respondents were assured that they could withdraw at any time without consequences.

## Conflicts of Interest

The authors declare no conflicts of interest.

## Transparency Statement

The corresponding author, Alpha Kargbo, affirms that this manuscript is an honest, accurate, and transparent account of the study being reported; that no important aspects of the study have been omitted; and that any discrepancies from the study as planned (and, if relevant, registered) have been explained.

## Data Availability

The data that support the findings of this study are available on request from the corresponding author. The data are not publicly available due to privacy or ethical restrictions.
